# P-2179. Nirsevimab in Patient Samples Does Not Interfere with Respiratory Syncytial Virus (RSV) Detection by Commercially Available Rapid Antigen Tests

**DOI:** 10.1093/ofid/ofae631.2333

**Published:** 2025-01-29

**Authors:** Sarah R Sincero, Katie Streicher, Elizabeth J Kelly, Kevin M Tuffy, Deidre Wilkins

**Affiliations:** AstraZeneca, Gaithersburg, Maryland; AstraZeneca, Gaithersburg, Maryland; AstraZeneca, Gaithersburg, Maryland; AstraZeneca, Gaithersburg, Maryland; Translational Medicine, Vaccines & Immune Therapies, BioPharmaceuticals R&D, AstraZeneca, Gaithersburg, MD

## Abstract

**Background:**

Nirsevimab is an extended half-life monoclonal antibody (mAb) that binds to the prefusion conformation of the respiratory syncytial virus (RSV) fusion (F) protein, and is approved for the prevention of RSV-associated lower respiratory tract infection in neonates and infants. Rapid antigen testing is routinely used for the clinical diagnosis of RSV, and primarily relies on direct interaction with the RSV F protein in patient nasal samples. While expected mAb levels in nasal samples are low (< 1 μg/mL), some mAbs targeting the RSV F protein, such as palivizumab, have been shown to interfere with RSV rapid antigen test results. We assessed whether the presence of nirsevimab in samples may interfere with RSV detection by rapid antigen tests, leading to false-negative or equivocal results.

**Figure 1.**
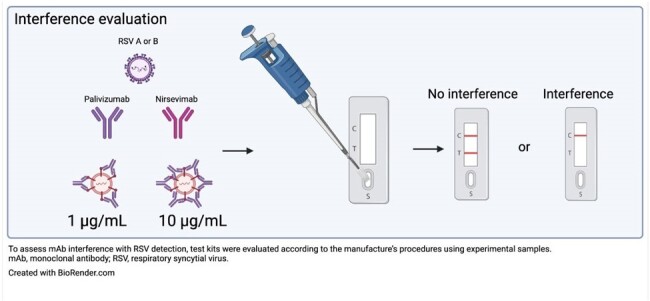
Evaluation of nirsevimab- and palivizumab-associated interference with RSV detection by rapid antigen tests

**Methods:**

We evaluated six waived RSV rapid antigen tests (simple tests with a low risk of erroneous results), representative of those in the US Food and Drug Administration Clinical Laboratory Improvement Amendments database. To assess mAb interference with RSV detection, each test kit was performed according to the manufacture’s procedures (Figure 1). Test samples contained phosphate-buffered saline spiked with RSV subtype A or B at 5 × 10^5^ plaque forming units/mL, and nirsevimab or palivizumab at two concentrations (1 or 10 µg/mL). For RSV-positive results, the intensity of the line on the test device was graded by observers as a ‘weak’ or ‘strong’ RSV detection signal.

**Table 1.**
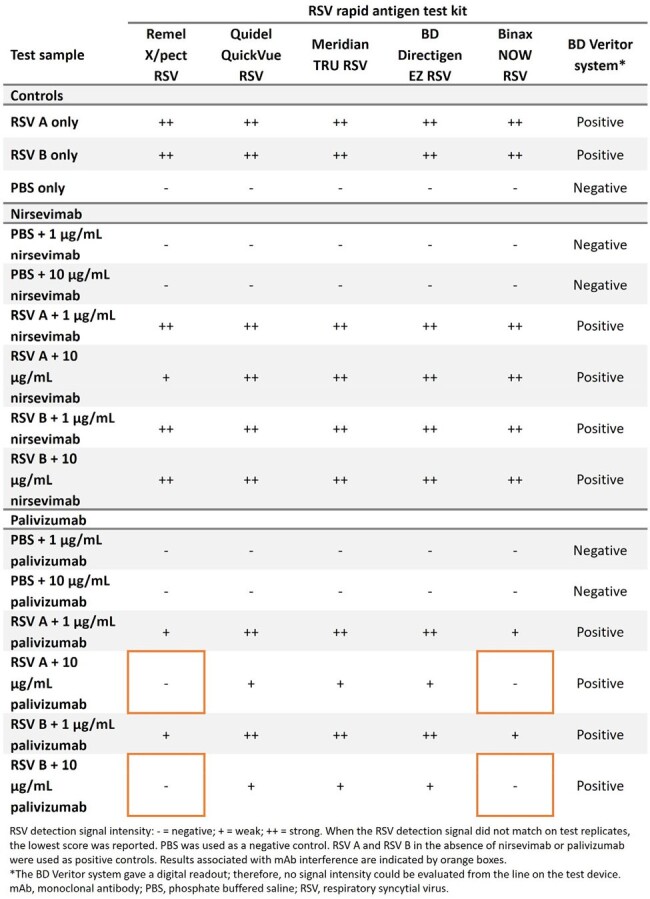
Effects of nirsevimab and palivizumab on RSV detection by a panel of rapid antigen test kits

**Results:**

RSV was detected by all rapid antigen tests in the presence of nirsevimab at both concentrations, with only a slight decrease in the RSV A detection signal observed for the Remel X/pect RSV kit at concentrations of 10 µg/mL (Table 1). RSV was not detected by the Remel X/pect RSV and Binax NOW RSV kits in samples with 10 µg/mL of palivizumab, and decreases in RSV detection signals were observed for the Quidel QuickVue RSV, Meridian TRU RSV, and BD Directigen EZ RSV kits (Table 1).

Funding statement

**Figure:**
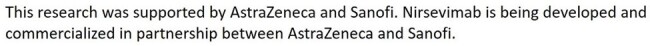

**Conclusion:**

Nirsevimab did not interfere with RSV detection for the six RSV rapid antigen tests assessed, even at a high concentration of 10 µg/mL. In contrast, 10 µg/mL of palivizumab did interfere with RSV detection for two test kits. Our findings suggest that patients who have received nirsevimab are unlikely to require alternative assays for clinical RSV diagnosis.

**Disclosures:**

Sarah R. Sincero, BSc, AstraZeneca: Employee of AstraZeneca and may own AstraZeneca stock or stock options. Katie Streicher, PhD, AstraZeneca: Employee of AstraZeneca and may own AstraZeneca stock or stock options. Elizabeth J Kelly, PhD, AstraZeneca: Former employee of AstraZeneca.|AstraZeneca: Stocks/Bonds (Public Company)|PLP: Stocks/Bonds (Public Company)|Sanofi: Current employee of Sanofi.|Sanofi: Stocks/Bonds (Public Company) Kevin M. Tuffy, MS, AstraZeneca: Employee of AstraZeneca and may own AstraZeneca stock or stock options. Deidre Wilkins, BSC, AstraZeneca: Employee of AstraZeneca and may own AstraZeneca stock or stock options.

